# Global research trends in penile cancer: Bibliometric and visualized analysis

**DOI:** 10.3389/fonc.2022.1091816

**Published:** 2023-01-06

**Authors:** Sheng Deng, Zhihua Xuan, Junlong Feng, Haisong Li, Bin Wang, Zhen Yang, Lihua Xuan, Fanchao Meng, Lu Wang, Yangchun Xiao, Jisheng Wang

**Affiliations:** ^1^ Department of Andrology, Shunyi Hospital, Beijing Hospital of Traditional Chinese Medicine, Beijing, China; ^2^ Department of Andrology, Dongzhimen Hospital, Beijing University of Chinese Medicine, Beijing, China; ^3^ Department of Urology Surgery, The Third Affiliated Hospital of Beijing University of Chinese Medicine, Beijing, China; ^4^ Department of Surgery, Beijing Xuanwu Traditional Chinese Medicine Hospital, Beijing, China; ^5^ Department of Emergency, Shunyi Hospital, Beijing Hospital of Traditional Chinese Medicine, Beijing, China

**Keywords:** penile cancer, bibliometrics, data visualization, research status, hotspots

## Abstract

**Background:**

Penile cancer is a malignant tumor of the genitourinary system that mostly occurs in middle-aged and elderly men aged 50–70 years, which can seriously affect physical, psychological, and sexual health. Hundreds of original articles and reviews on penile cancer are published each year. However, a bibliometric analysis of these publications has not been performed.

**Objective:**

This study aimed to systematically analyze and visualize penile cancer-related publications through bibliometrics and reveal identified topics, hotspots, and knowledge gaps in related fields.

**Methods:**

Based on the Web of Science core collection database, we first analyzed the quantity and quality of publications in the field of penile cancer. Second, we profiled the publishing groups in terms of country, institution, author’s publication, and cooperation network. Then, we systematized and summarized the hot topics of research.

**Results:**

This bibliometric analysis was conducted from 2001 to 2022. The analysis identified 1,687 articles and reviews, which were published in 432 journals. The number of publications and citations on penile cancer-related research has steadily increased over the last two decades. Furthermore, academic institutions in Europe and the United States play a leading role in penile cancer research. The country, institution, journal, and author with the most publications were the United States (507), H Lee Moffitt Cancer Research Center (96), Journal of Urology (83), and Spiess P (87), respectively. The most frequently used keywords were penile cancer (743), squamous-cell carcinoma (717), cancer (380), carcinoma (232), lymphadenectomy (229). 16 keyword clustering information was obtained, including #0 male circumcision, #1 lichen sclerosus, #2 chemotherapy, #3 penile neoplasms, #4 targeted therapy, #5 resection margin, #6 cervical cancer, #7 lymph node dissection, #8 prognostic factor, #9 prostate cancer, #10 inguinal lymph node dissection, #11 human papillomavirus DNA, #12 gene, #13 penile intraepithelial neoplasia, #14 male sexual function, and #15 penile cancer.

**Conclusion:**

More and more scholars are devoted to the research on penile cancer. This bibliometric analysis revealed that the main research topics and hotspots in penile cancer included risk factors and surgical treatment plans.

## 1 Introduction

Penile cancer is a malignant tumor of the genitourinary system that mostly occurs in middle-aged and elderly men aged 50–70 years, which can seriously affect physical, psychological, and sexual health. Due to different countries, ethnic groups, religious beliefs, and health habits, the incidence of penile cancer has obvious regional differences. Penile cancer accounts for 0.4%–0.6% of all male malignancies in Europe and the United States (USA) (1, 2). Penile cancer is a serious public health problem in economically underdeveloped parts of Asia, Africa, and South America, where it can account for up to 10% of cases (3).

Penile cancer is classified using the tumor, node, metastases (TNM) staging system. Because the penis consists of different types of cells, different types of cancer can occur (4). The majority of tumors (over 95%) are squamous cell carcinomas (SCC), of which there are several recognized subtypes: warty, papillary, basaloid, verrucous, and sarcomatoid. Other non-SCC cancer types include sarcomatoid tumors, malignant melanoma, extramammary Paget’s disease, and malignant lymphomas (5).

The etiology and pathogenesis of penile cancer remain unclear, and its possible risk factors include phimosis, smoking, human immunodeficiency virus (HIV), human papillomavirus (HPV), chronic penile inflammation, and a history of impurity (6–8). Surgery is the main treatment for penile cancer, but there is no consensus on the proportion of lesion removal and penile loss. Early penile cancer without metastasis can be completely cured by surgical resection of the lesion, with a 5-year survival rate of up to 90%. However, once the tumor progresses to inguinal lymph node metastasis, the 5-year survival rate decreases to 50% (9). Additionally, radiotherapy, chemotherapy, and targeted therapy are alternative treatment options for penile cancer, but there is still a risk of local recurrence (10). Doctors must closely monitor these patients through follow-up.

Bibliometrics is the quantitative analysis of literature, which is widely used for evaluating research trends and hotspots in various fields (11). VOSviewer and CiteSpace software is commonly used in bibliometrics for co-word analysis, co-citation analysis, and literature coupling analysis, being able to visually display the outcomes. This software has the advantage of clustering technology and map presentation. It can rapidly analyze research trends in a certain field and exhibit them in the form of multivariate integrated visual knowledge maps (12, 13).

Hundreds of original articles and reviews on penile cancer are published each year. However, so far, there has been no systematic analysis of penile cancer-related publications. Therefore, this study aimed to systematically analyze and visualize penile cancer publications over the past 20 years using related bibliometric software, such as VOSviewer and CiteSpace. Furthermore, the study aimed to summarize the achievements attained in this field, understand the research direction and hotspot areas, and provide a reference for future studies.

## 2 Methods

### 2.1 Ethics statement

The present study did not involve any human subject participation, and it was entirely performed using the bibliometric data retrieved from the Web of Science database (WOS, https://www.webofscience.com/wos/woscc/basic-search). Hence, it was deemed to be exempted from the Institutional Review Board approval.

### 2.2 Data sources and collection

The WOS database is the most commonly used and widely accepted database in scientific or bibliometric research. It contains nearly 9,000 high-impact journals and more than 12,000 academic conference proceedings, which provide a comprehensive overview of research in the scientific, technological, and medical research fields (14, 15).

Publications on penile cancer were retrieved on October 02, 2022. The time span was set between January 01, 2001, and October 01, 2022. First, the “WOS Core Collection” was selected on the search page. Article types were refined into “article” and “review.” “Plain text” was chosen for the file format, while “Full Record and Cited References” was chosen for the record content.

The search query string was described as follows: “penis cancer” (topic), “penis tumor” (topic), or “penis tumor” (topic) and “penis oncology” (topic), “penis neoplasm” (topic), “penis carcinoma” (topic), “penile cancer” (topic), “penile tumor” (topic), “penile tumor” (topic), “penile oncology” (topic), “penile neoplasm” (topic), or “penile carcinoma” (topic) and article or review article (document types).

### 2.3 Data analysis

The data were downloaded and analyzed by two researchers to ensure the accuracy of the data and the repeatability of the research. Microsoft Excel 2019 and GraphPad Prism 7 were applied to analyze the targeted files and exported the line charts and tables of top-cited or productive countries/regions, institutions, authors, journals, references, and keywords.

The test of fit (R^2^) was used to predict the relationship between publication year and publication output to compare the degree of agreement between the predicted results and the actual occurrence. The closer R^2^ is to 1, the better the fitting degree of the regression line to the observed value is; otherwise, the worse it is. The quantity and quality of academic production were evaluated by H-index proposed by Hirsch. Total link strength (TLS) was defined as the total number of co-occurrences. At the same time, the 2021 version of the Impact factor (IF) and Journal impact factor (JIF) quartile, as important indicators to measure the scientific value of research, were also included in the analysis. To characterize the nature of a cluster, CiteSpace was based on three special algorithms, namely log likelihood ratio (LLR), late semantic indexing (LSI), and mutual information (MI). Of the three, LLR generally gives the best results in terms of the uniqueness and coverage of cluster-related topics. Therefore, LLR was used for keyword clustering in this study.

### 2.4 Bibliometric analysis and visualization software

CiteSpace (https://citespace.podia.com/download, R6.1.3) is a visual analysis software widely used in scientific papers. It is based on scientometric data and information visualization technology. Further, it presents the knowledge structure by analyzing the underlying knowledge, patterns, and distribution of the literature. In this study, CiteSpace was used for keyword clustering and burst word analysis (16).

VOSviewer (https://www.vosviewer.com/, R1.6.18) is a bibliometric analysis software for mapping knowledge. It can be used for co-word analysis, co-citation analysis, coupling analysis of documents, and result visualization. In this study, VOSviewer was used to visualize countries, authors, institutional collaborations, cited journals, and keyword co-occurrences and construct density maps (17).

The FoamTree function of Carrot2 (https://search.carrot2.org, R3.10.3) is used to visually display the topic categories. In this study, Carrot2 was utilized to extract keywords of significance and give relative impact to each keyword (18).

## 3 Results

### 3.1 Analysis of global publishing trends

**Figure 1:**
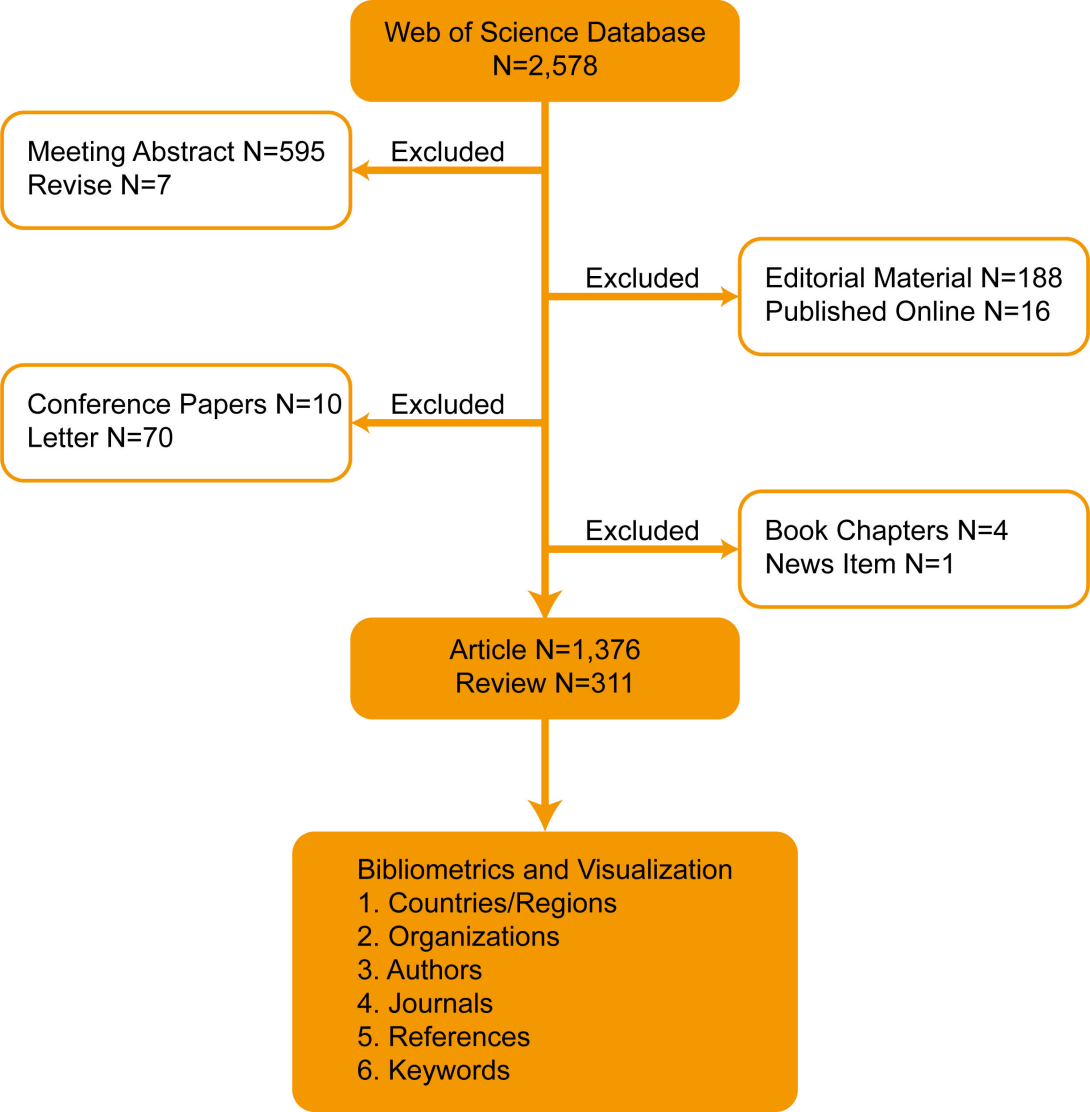
Flowchart of the search strategy.

Analysis of the annual number of published papers is important as it reflects the growth of knowledge in a certain field. In this study, 1,687 articles and reviews on penile cancer research were selected out of the 2,578 records retrieved from the Web of Science database. A total of 891 conference abstracts, letters, news, book chapters, and editorial materials were excluded (**Figure 1**).

**Figure 2:**
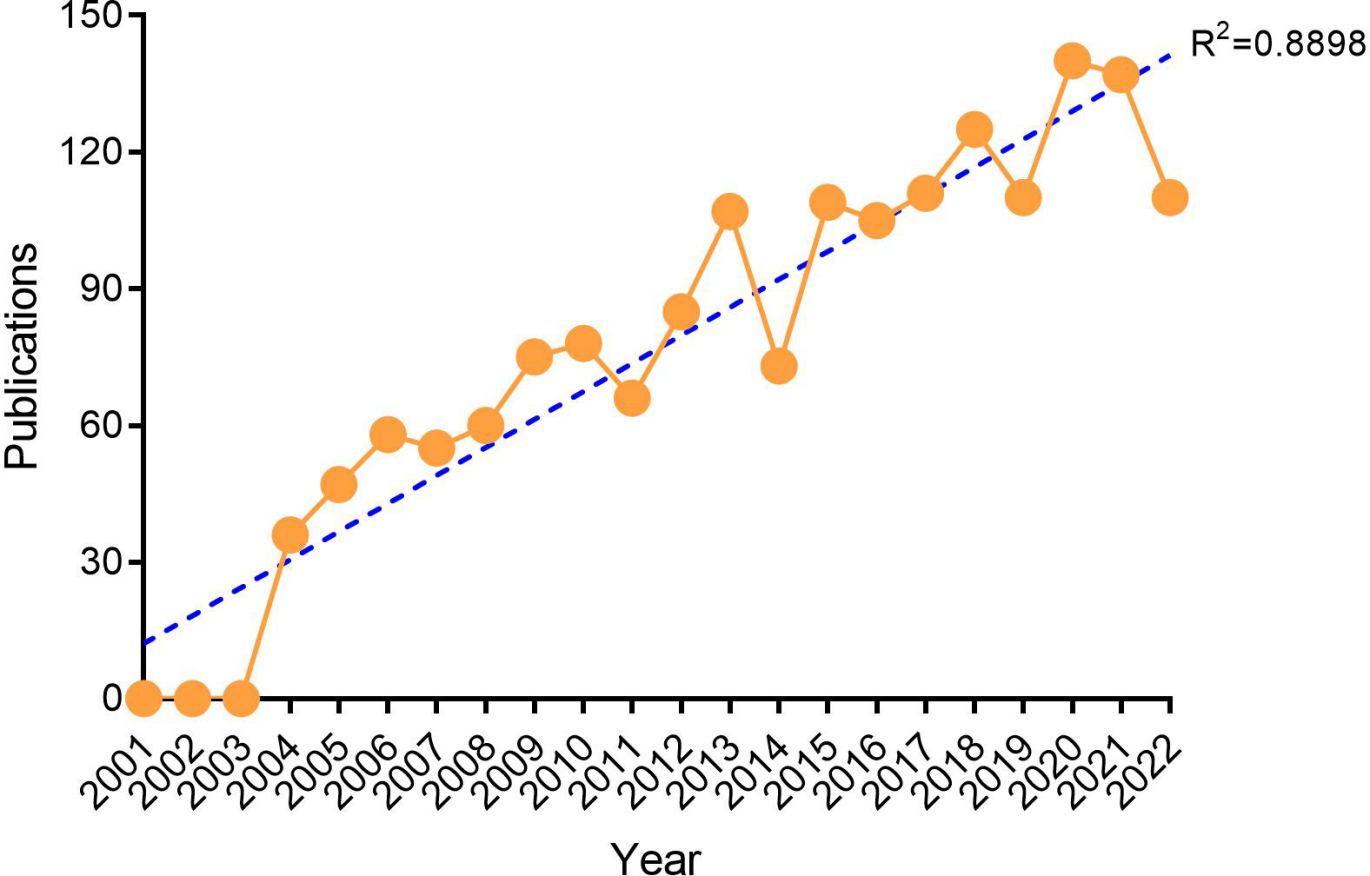
Annual trends of global publications.

Although the time span of our search was from January 1, 2001, to October 1, 2022, no papers that meet both the search term and the article type could be included before 2004. This study showed that from 2004 to 2011, the number of publications on penile cancer increased slowly. The turning point occurred in 2012. Since then, the number of papers published has increased rapidly. The number of papers published each year exceeded 100, reaching a record high (140) in 2020. This bibliometric analysis generally showed a linear growth trend (R^2^ = 0.8898) in penile cancer-related research, reflecting the increasing interest in this research field (**Figure 2**).

### 3.2 Analysis of distribution and cooperation of leading countries/regions

**Figure 3:**
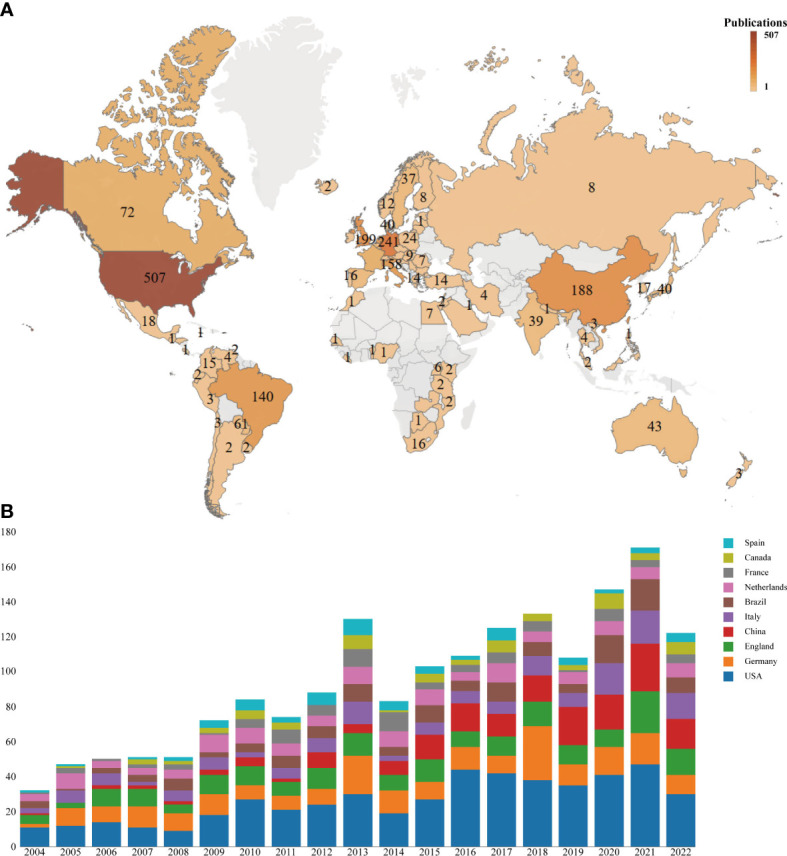
Global analysis of the research trends in penile cancer. **(A)** World map based on the total publications of different countries/regions. **(B)** The changing trend of the annual publication quantity in the top 10 countries/regions from 2004 to 2022.

A total of 84 countries/regions published papers on penile cancer. The USA had the largest number of publications (507, 30.05%), followed by Germany (241, 14.29%), England (199, 11.80%), China (188, 11.14%), and Italy (158, 9.37%) (**Table 1**, **Figure 3**). Additionally, the H index (52) and total citations (11954) of the USA ranked first, followed by England (44, 8170), Netherlands (48, 6478), Germany (30, 4691), and Italy (33, 4106) (**Table 1**). The results showed that the above-mentioned countries were more interested in the related research of penile cancer.

**Table 1:** Top 10 productive countries/regions.

Rank	Countries/Regions	Publications	Percentage	H-index	Citations
1	USA	507	30.05	52	11,954
2	Germany	241	14.29	30	4,691
3	England	199	11.80	44	8,170
4	China	188	11.14	24	2,032
5	Italy	158	9.37	33	4,106
6	Netherlands	140	8.30	48	6,478
7	Brazil	140	8.30	27	2,395
8	France	91	5.39	27	3,937
9	Canada	72	4.27	22	1,845
10	Spain	68	4.03	27	3,984

**Figure 4:**
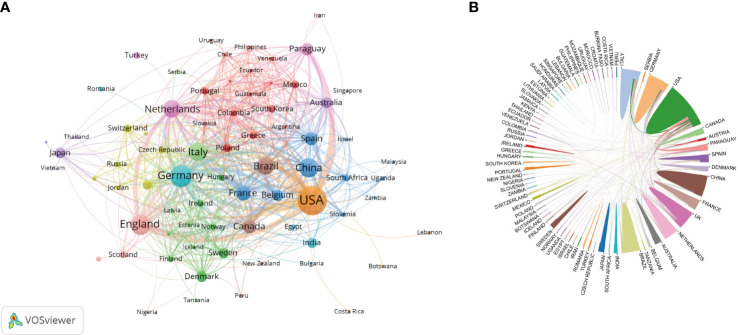
Co-occurrence map of countries/regions. **(A)** The size of the nodes represents the number of articles; the thickness of the curve represents the strength of the collaboration; the colors represent different collaboration groups. **(B)** The thickness of the line between countries reflects the frequency of the cooperation.

VOSviewer was used to analyze the cooperation of different countries. The line between nodes indicates the co-authorship between countries; the thicker the line, the stronger the cooperative relationship. The results showed that the USA, Germany, Italy, and England had more cooperation with other countries. However, cooperation between other countries was weaker (**Figure 4**).

### 3.3 Analysis of distribution and cooperation of leading institutions

A total of 2,266 institutions were involved in publishing penile cancer-related papers. The top five institutions with the highest number of publications were H Lee Moffitt Cancer Research Center (96), Antoni Van Leeuwenhoek Hospital (60), Fudan University (45), Netherlands Cancer Institute (44), and University Texas Md Anderson Cancer Center (43) (**Table 2**). The top five institutions with the highest number of total citations were Antoni Van Leeuwenhoek Hospital (3,454), H Lee Moffitt Cancer Research Center (1,526), Harvard University (1,397), University Texas Md Anderson Cancer Center (1,196), and Netherlands Cancer Institute (1,163) (**Table 2**).

**Table 2:** Top 10 productive institutions.

Rank	Organizations	Publications	Original country	Citations	TLS
1	H Lee Moffitt Cancer Research Center	96	USA	1,526	180
2	Antoni Van Leeuwenhoek Hospital	60	Netherlands	3,454	64
3	Fudan University	45	China	737	96
4	Netherlands Cancer Institute	44	Netherlands	1,163	85
5	University Texas Md Anderson Cancer Center	43	USA	1,196	74
6	University of Florida	38	USA	947	77
7	University College London Hospital	37	UK	987	70
8	Sun Yat-Sen University	30	China	341	73
9	University of Sao Paulo	28	Brazil	313	36
10	Harvard University	27	USA	1,397	107

**Figure 5:**
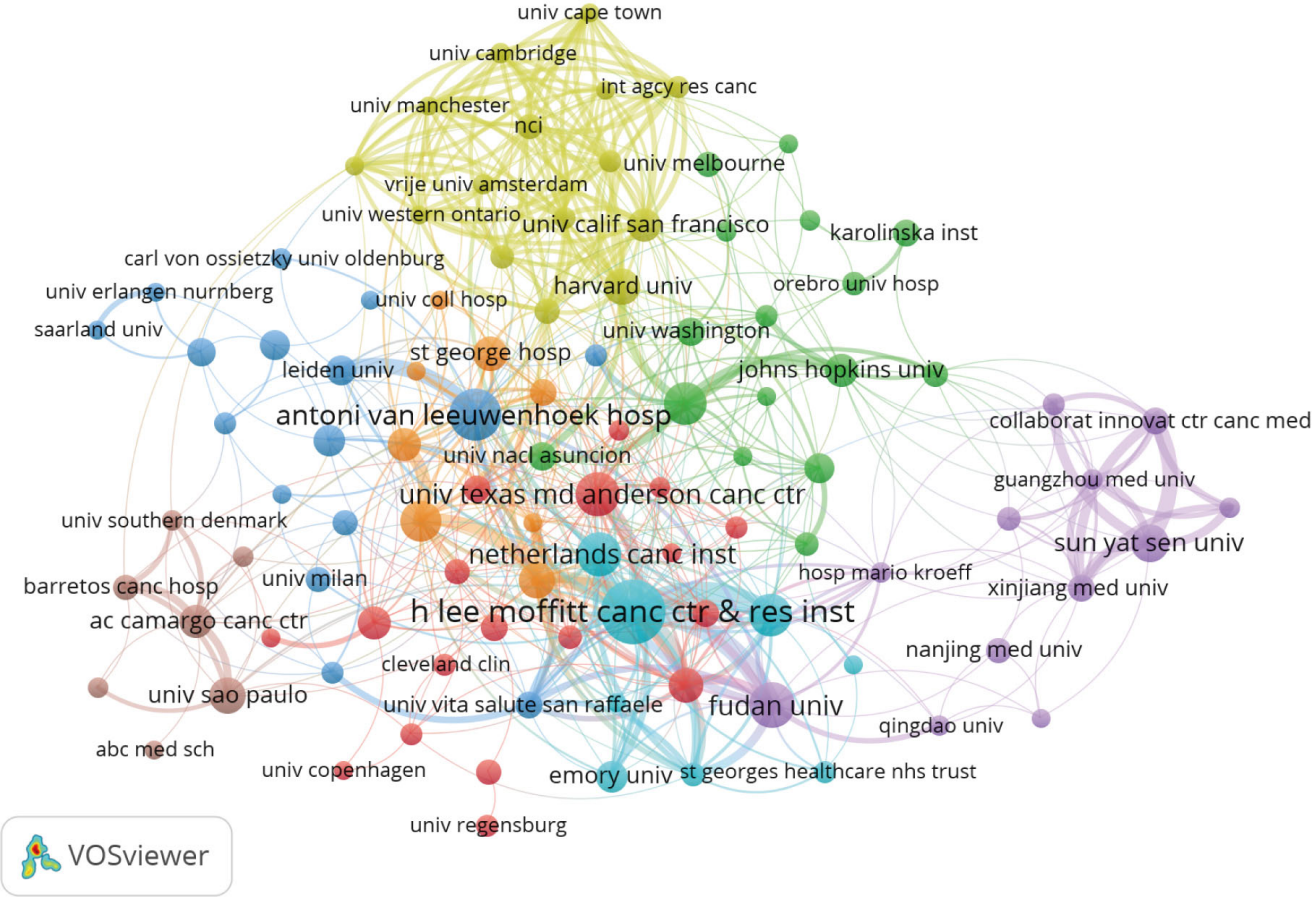
Co-occurrence map of institutions. The size of the nodes represents the number of articles; the thickness of the curve represents the strength of the collaboration; the colors represent different collaboration groups.

Antoni Van Leeuwenhoek Hospital, H Lee Moffitt Cancer Research Center, and University Texas Md Anderson Cancer Center were at the center of the partnerships. However, most institutions were fragmented and lacked cooperation. The overall network density was low (density = 0.0078) (**Figure 5**).

### 3.4 Analysis of authors and co-cited authors

The author co-occurrence analysis identified the core authors in penile cancer-related research and the strength of collaboration between authors. Co-cited analysis means that when two authors or papers are cited by a third author or paper at the same time, the two authors or papers have a co-cited relationship.

**Figure 6:**
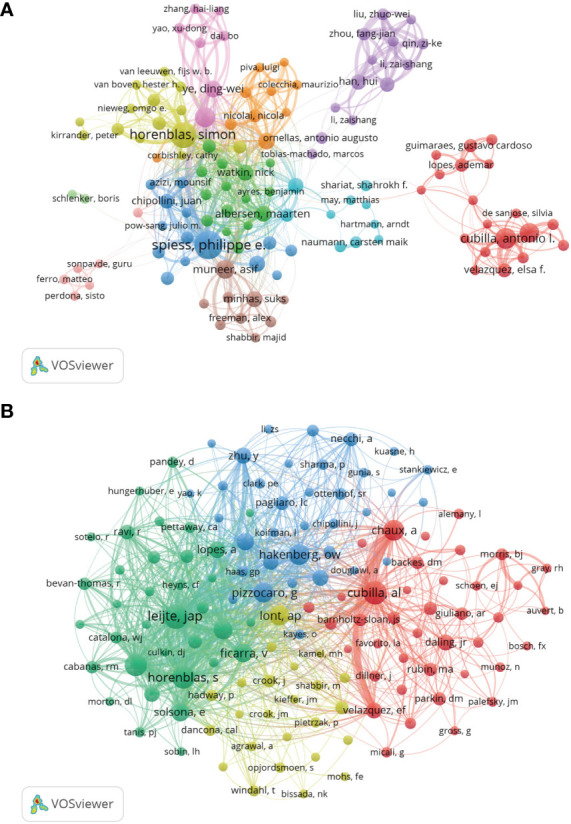
Analysis of authors and co-cited authors. **(A)** Co-occurrence map of Authors. The size of the nodes represents the number of articles. **(B)** Co-cited authors analysis map. The size of the nodes represents the number of co-citations.

This analysis revealed a total of 7,290 authors and 19,136 co-cited authors. Among them, Spiess P (87), Horenblas S (71), Cubilla A (46), Zhu Y (40), and Chaux A (37) had the highest number of publications (**Table 3** and **Figure 6A**). The author’s cooperation represents teamwork, but the cooperation among teams is less, and the research is relatively fragmented. The co-citation analysis showed that Leijte J (611), Cubilla A (553), Horenblas S (507), Kroon B (484), and Chaux A (427) had the most co-citations (**Table 3** and **Figure 6B**). The results showed that the above-mentioned authors were more interested in the related research of penile cancer.

**Table 3:** Top 10 productive authors and co-cited authors.

Rank	Authors	Publications	Citations	Rank	Co-Cited Authors	Co-Citations
1	Spiess P	87	1,279	1	Leijte J	611
2	Horenblas S	71	3,324	2	Cubilla A	553
3	Cubilla A	46	1,168	3	Horenblas S	507
4	Zhu Y	40	660	4	Kroon B	484
5	Chaux A	37	1,040	5	Chaux A	427
6	Muneer A	35	688	6	Hakenberg O	427
7	Necchi A	28	786	7	Pizzocaro G	401
8	Ye D	27	410	8	Lont A	381
9	Albersen M	26	182	9	Ornellas A	349
10	Minhas S	25	1,057	10	Ficarra V	348

### 3.5 Analysis of leading journals and co-cited journals

All the selected papers were published in 432 journals. The top five high-yield journals were the Journal of Urology (83), BJU International (82), Urologic Oncology-Seminars and Original Investigations (81), Urology (58), and Urologe (50). The most cited journals were the Journal of Urology (3,534), European Urology (3,372), BJU International (2,308), Urology (1,305), and World Journal of Urology (960) (**Table 4**).

**Table 4:** Top 10 productive journals.

Rank	Journals	Publications	Citations	IF (2021)	JIF quartile
1	Journal of Urology	83	3,534	7.60	Q1
2	BJU International	82	2,308	5.969	Q1
3	Urologic Oncology-Seminars and Original Investigations	81	343	2.954	Q2
4	Urology	58	1,305	2.633	Q2
5	Urologe	50	166	0.803	Q3
6	European Urology	43	3,372	24.267	Q1
7	World Journal of Urology	35	960	3.661	Q1
8	Translational Andrology and Urology	32	276	2.479	Q3
9	Current Opinion in Urology	29	227	2.808	Q2
10	International Braz J Urol	26	356	3.05	Q2

Analysis of the co-cited journals showed that 4,804 journals were co-cited. The top five co-cited journals were the Journal of Urology (6,476), European Urology (3,257), BJU International (2,446), Urology (1,879), and International Journal of Cancer (1,162) (**Table 5**). Most of the top ten productive and co-cited journals are divided into Q1 and Q2, reflecting the outstanding academic contributions of penile cancer-related research.

**Table 5:** Top 10 co-cited journals.

Rank	Co-Cited Journals	Co-Citations	IF (2021)	JIF quartile
1	Journal of Urology	6,476	7.60	Q1
2	European Urology	3,257	24.267	Q1
3	BJU International	2,446	5.969	Q1
4	Urology	1,879	2.633	Q2
5	International Journal of Cancer	1,162	7.316	Q1
6	Journal of Clinical Oncology	1,040	50.717	Q1
7	American Journal of Surgical Pathology	998	6.298	Q1
8	World Journal of Urology	854	3.661	Q1
9	New England Journal of Medicine	732	176.079	Q1
10	Urologic Oncology-Seminars and Original Investigations	721	2.954	Q2

**Figure 7:**
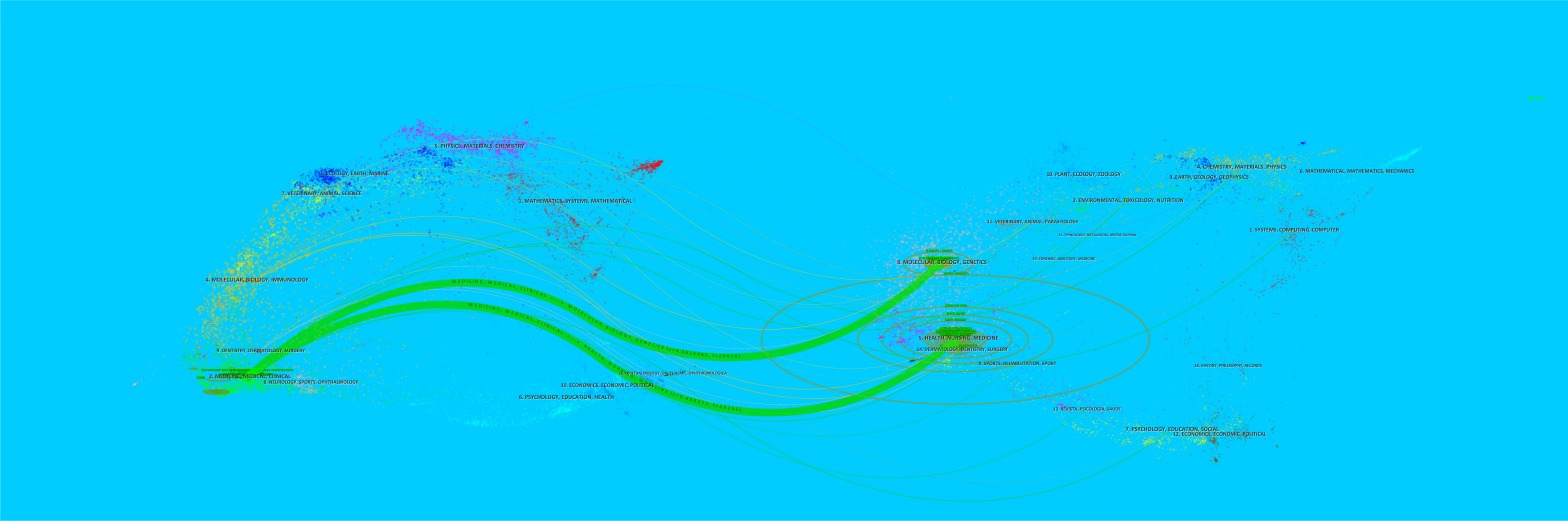
The dual-map overlay of journals in penile cancer. The more papers the journal publishes, the longer the vertical axis of the ellipse, and the greater the number of authors, the longer the horizontal axis of the ellipse.

Knowledge flow analysis is used to explore the evolutionary relationship of knowledge citations and co-citations between citing and cited journals. The dual-map overlay of journals can intuitively show the distribution of journals in various disciplines, the evolution of citation trajectories, and the drift of scientific research centers. The citing map is on the left and the cited map is on the right. In **Figure 7**, the two green citation paths indicate that the research of molecular, biology, genetics, or health, nursing, medicine journals is often cited by medicine, medical, clinical journals.

### 3.6 Analysis of references co-occurrence and burst references

**Figure 8:**
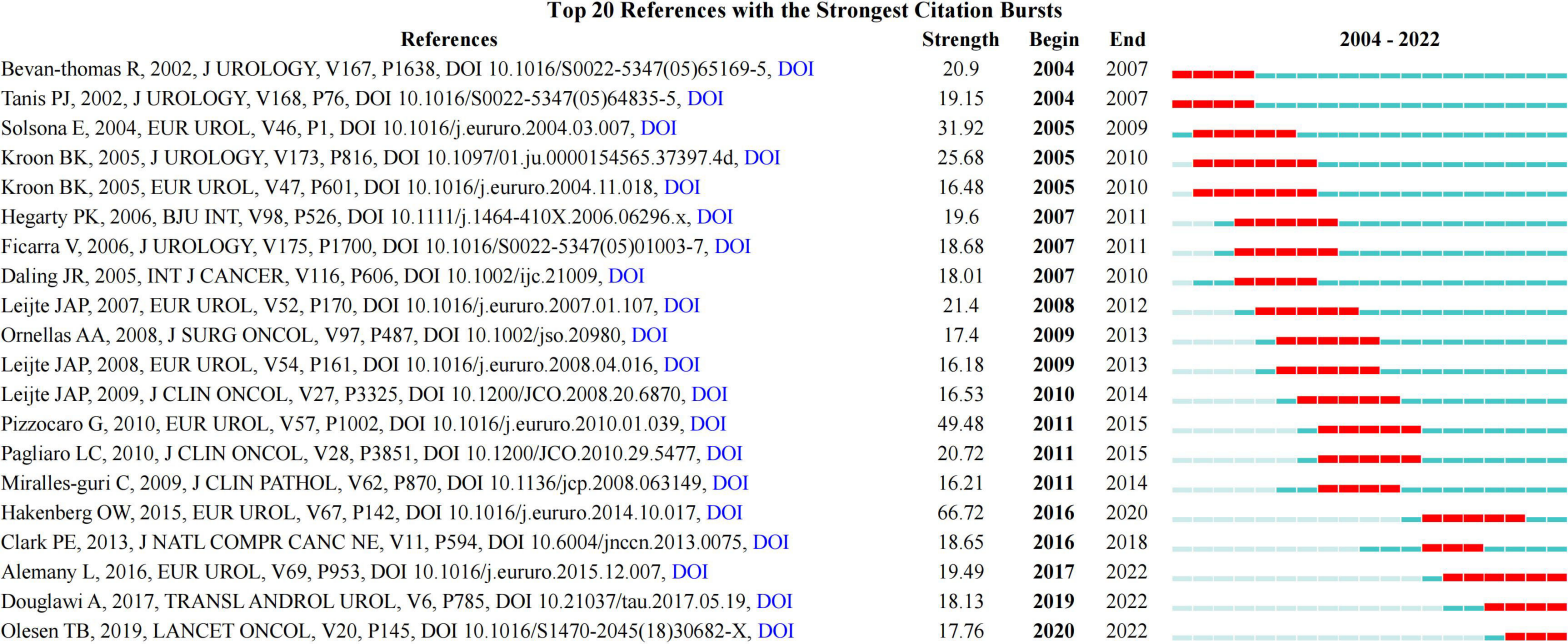
References burst analysis by CiteSpace.

A total of 1,687 references were obtained, of which three references Forman (2012), zur Hausen (2009), and Parkin (2006) were cited more than 900 times (**Table 6**). Additionally, a total of 20 references had the strongest citation bursts. The three references with the highest strength were Hakenberg O, 2015 (66.72), Pizzocaro G, 2010 (49.48), and Solsona E, 2004 (31.92). The first reference that triggered a citation burst appeared in 2004 (Bevan-Thomas R, 2002) (**Figure 8**).

**Table 6:** Top 10 cited references.

Title	Journals	Authors	Year	Citations
Global burden of human papillomavirus and related diseases	Vaccine	Forman D	2012	1,003
Papillomaviruses in the causation of human cancers - a brief historical account	Virology	zur Hausen H	2009	953
The burden of HPV-related cancers	Vaccine	Parkin D	2006	929
EAU Guidelines on Penile Cancer: 2014 Update	European Urology	Hakenberg O	2015	349
HPV prophylactic vaccines and the potential prevention of noncervical cancers in both men and women	Cancer	Gillison M	2008	331
Penile cancer: importance of circumcision, human papillomavirus and smoking in *in situ* and invasive disease	International Journal of Cancer	Daling J	2005	296
Systematic review of human papillomavirus prevalence in invasive penile cancer	Cancer Causes and Control	Backes D	2009	265
EAU Penile Cancer Guidelines 2009	European Urology	Pizzocaro G	2010	247
Male circumcision	Pediatrics	Blank S	2012	239
Patients with penile carcinoma benefit from immediate resection of clinically occult lymph node metastases	Journal of Urology	Kroon B	2012	226

### 3.7 Analysis of keyword co-occurrence, clustering, and burst term

**Figure 9:**
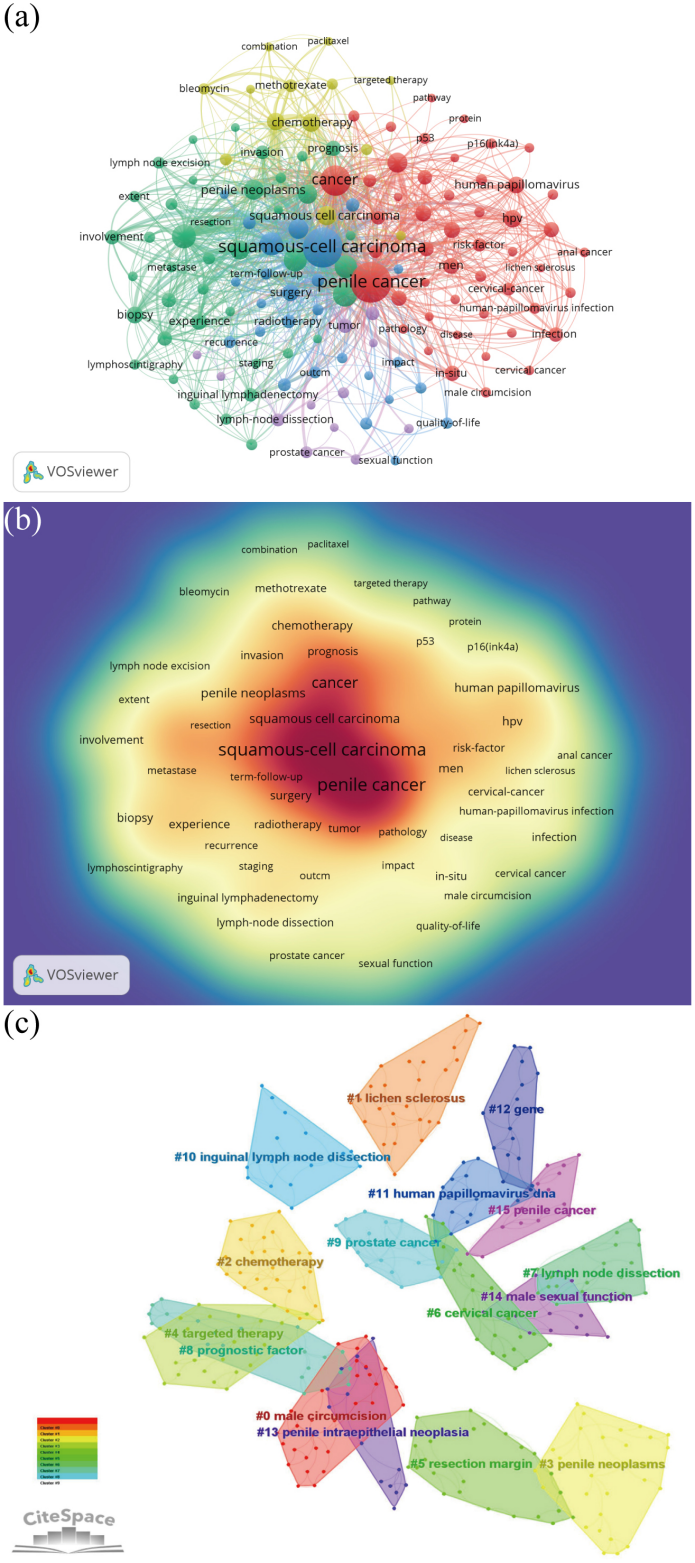
Keyword analysis. **(A)** Keyword co-occurrence analysis map obtained using VOSviewer. The size of the nodes represents the number of occurrences; the thickness of the curve represents the strength of collaboration; the different colors represent the different clusters. **(B)** Keyword density visualization analysis. The higher the intensity of the red color node, the higher the number of keywords. **(C)** Keyword clustering map analysis through CiteSpace. A total of 16 categories of keywords were obtained. The different color blocks represent different keyword clusters.

Keywords frequently reflect an article core and the main content. The cluster map reflects research hotspots effectively. A total of 4,149 keywords were obtained. The top ten keywords obtained were penile cancer (743), squamous-cell carcinoma (717), cancer (380), carcinoma (232), lymphadenectomy (229), survival (206), management (206), penile carcinoma (191), prognostic-factor (160), and human papillomavirus (160) (**Figures 9A, B**). After clustering through CiteSpace software, a total of 16 clustering words were obtained, namely: #0 male circumcision, #1 lichen sclerosus, #2 chemotherapy, #3 penile neoplasms, #4 targeted therapy, #5 resection margin, #6 cervical cancer, #7 lymph node dissection, #8 prognostic factor, #9 prostate cancer, #10 inguinal lymph node dissection, #11 human papillomavirus DNA, #12 gene, #13 penile intraepithelial neoplasia, #14 male sexual function, and #15 penile cancer (**Figure 9C**).

**Figure 10:**
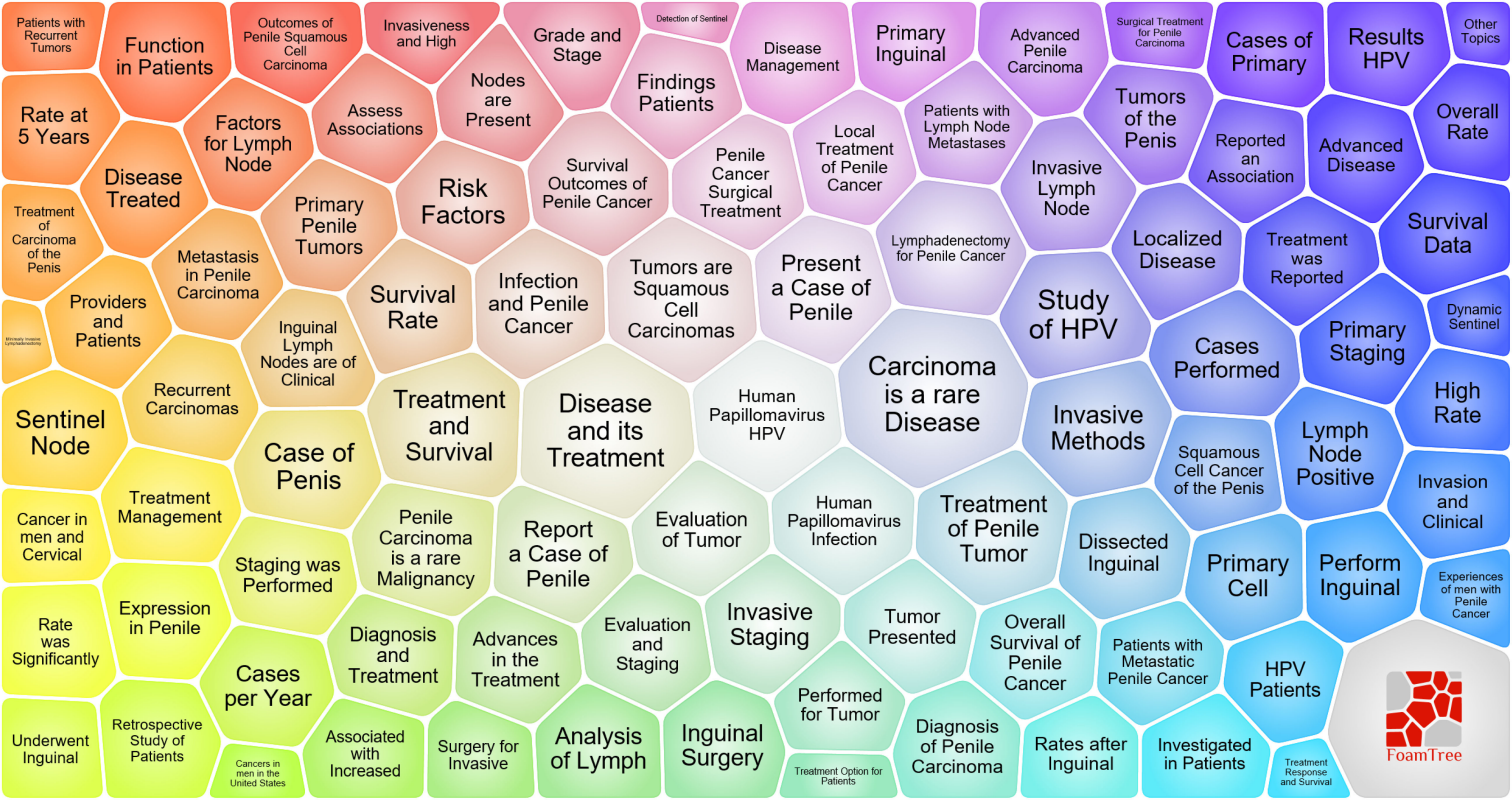
Topic categories survey for penile cancer based on the carrot 2.

Using the FoamTrees function of Carrot2 and taking keywords as the data source, 100 clustering results were obtained (**Figure 10**). The larger the area of foam, the higher the heat of the study. It can be seen that “carcinoma is a ray disease,” “disease and its treatment,” “tumors are squamous cell carcinomas,” “treatment of penile tumor,” “treatment and survival,” “lymphadenectomy for penile cancer,” and “human papillomavirus HPV” were the main topics related to penile cancer.

**Figure 11:**
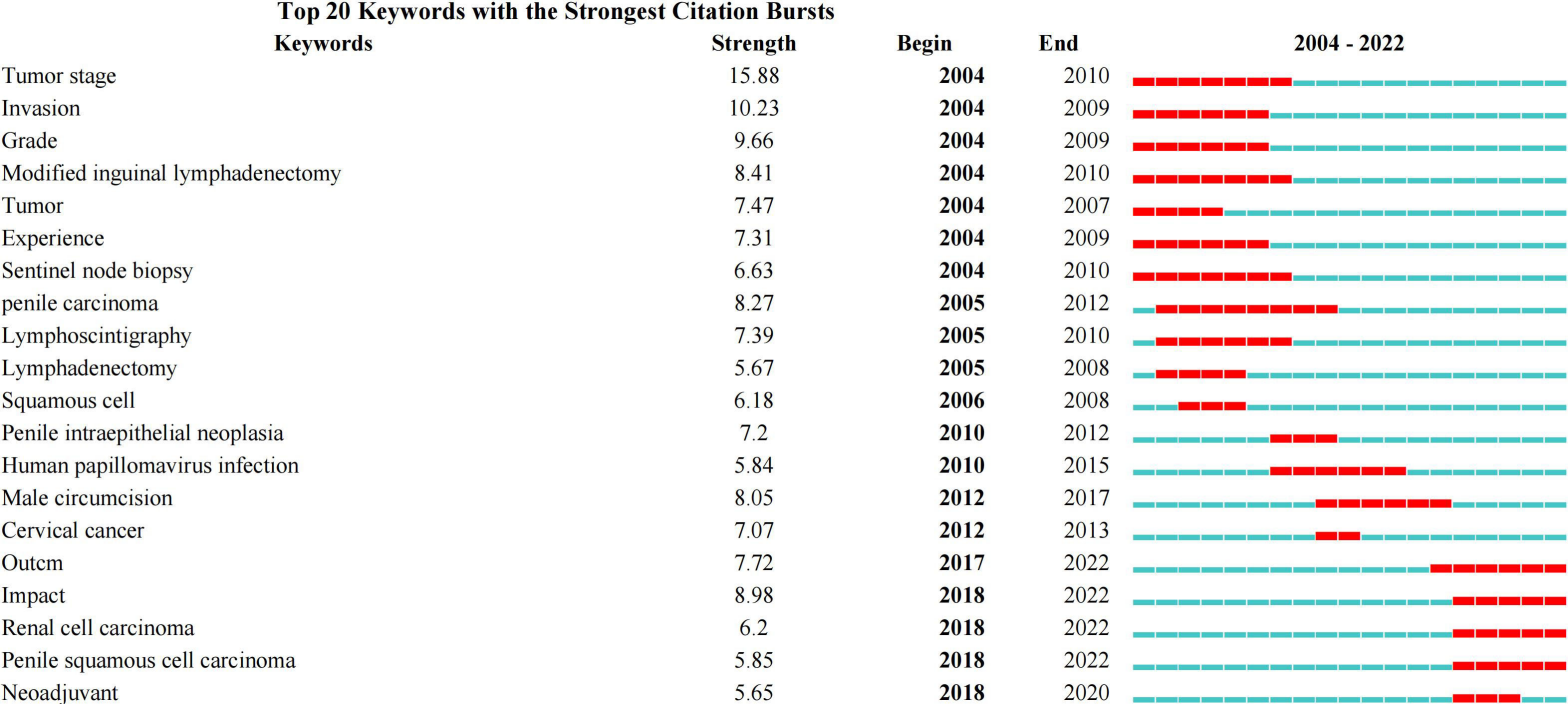
Keywords burst analysis by CiteSpace.

Furthermore, a total of 20 keyword burst term analysis results were obtained. From 2004 to 2008, research on penile cancer focused on tumor staging, invasion, and surgical classification. From 2010 to 2017, the research related to penile cancer in this stage was transformed into research on human papillomavirus infection, male circulation, and other etiology and prevention measures. Since 2017, research on penile cancer has begun to explore the development of neoadjuvants on the basis of previous studies. The three burst words with the highest strength were tumor stage (15.88), invasion (10.23), and grade (9.66), **Figure 11**.

## 4 Discussion

With the advent of big data, researchers need to understand the developments within penile cancer-related research. The bibliometric analysis uses visualization software, such as VOSviewer and CiteSpace, to comprehensively analyze the existing literature, understand the research trends, and predict future research hotspots (19). This study was the first bibliometric analysis of the related publications on penile cancer in the past 20 years.

### 4.1 General information on penile cancer-related literature

In the past 20 years, the number of papers published in the research related to penile cancer has shown a linear upward trend (R^2^ = 0.8898). Especially in the past five years, the number of papers published each year exceeded 110, indicating that researchers in various countries are more interested in the field of penile cancer. It can be predicted that more countries and researchers will participate in penile cancer research in the future. Additionally, most studies were funded by the National Science Foundation and large pharmaceutical enterprises, showing that, at the national scientific and technological levels, more attention has been paid to research in this field.

Compared with other countries, the USA ranked first in the number of publications, citations, and H-index, being the major contributor in this field. It was followed by Germany, England, China, and Italy. It can be seen that the top five countries in terms of productivity were mainly concentrated in Europe and the USA. Although the incidence rate of penile cancer in some Asian, African, and South American countries is significantly higher than that in European and American countries, only China and Brazil ranked in the top 10, and the H-index of both countries was low, indicating that the quality of published papers was not high. The possible reason is the lack of international cooperation, subject support, and language barriers between researchers. Among the top 10 institutions that published papers, only Fudan University, Sun Yat Sen University, and University of Sao Paulo came from Asia and South America, and the rest came from Europe and the USA. Most institutions showed little cooperation, and the network density was only 0.0078. Therefore, it is necessary to strengthen exchanges and cooperation between research institutions and countries, especially in Asia and South America.

IF, JIF quartile, and total citation are effective indicators to appraise journal quality. The top 3 journals for productivity were the Journal of Urology (83; 3534; IF = 7.60; Q1), BJU International (82; 2308; IF = 5.969; Q1), and Urologic Oncology-Seminars and Original Investigations (81; 343; IF = 2.954; Q2). Additionally, although the number of papers published by European Urology was low, its total citations and co-citations were higher than that of most journals, indicating the important influence of European Urology in this field. Most of the top 10 journals were divided into Q1 and Q2, reflecting outstanding academic contributions to penile cancer research. It is a new method to show the flow of knowledge at the journal level on the dual-map overlay of journals. All colored curves originating from the citing map and pointing to the ones cited are the citation connection lines, which completely show the ins and outs of the citation. In **Figure 7**, the two green citation paths indicate that the research of molecular, biology, genetics, or health, nursing, medicine journals is often cited by medicine, medical, clinical journals.

Spiess P had the most publications (87), followed by Horenblas S (71), Cubilla A (46), Zhu Y (40), and Chaux A (37). Furthermore, Horenblas S was cited the most. The main research direction of Spiess P was the surgical treatment of penile cancer and the management of patients with lymphatic metastasis (20, 21). The papers cited most frequently by Horenblas S mainly involved clinical guidelines, epidemiology, pathogenesis, and prevention of penile cancer (22, 23). Forman (2012), zur Hausen (2009), and Parkin (2006) all cited more than 900 times. Interestingly, these three references were about the relationship between HPV and penile cancer. Thus, it can be seen that researchers all over the world have a strong interest in the etiology of penile cancer (24–26).

### 4.2 Hotspots and frontiers of penile cancer research

Through the analysis and comparison of the above-discussed general information, we can see that the most influential authors and references were mostly review articles and clinical guides from internationally renowned institutions and journals. Combined with the co-occurrence, clustering, and burst analysis of keywords, we preliminarily identified the risk factors and surgical treatment plan of penile cancer as the main research topic and hotspot in this field.

#### 4.2.1 Risk factors of penile cancer

In recent years, people have had more knowledge about the risk factors of penile cancer, although the exact cause is still unclear. Penile cancer has two different pathogenic pathways. The first is related to high-risk HPV infection, and the second is related to chronic irritation and inflammation (27). HPV infection is a major risk factor for sexually transmitted infection of penile cancer (24). The HPV subtypes most frequently associated with penile cancer are HPV-16 and HPV-18. Studies have found that the carcinogenic pathogenesis of HPV infection is viral proteins E6 and E7-induced overexpression of cyclin-dependent kinase inhibitor 2A (p16^Ink4a^), which plays a role in cancer promotion, followed by the induction of malignant cell proliferation and carcinogenesis (28). Ferreux et al. have studied 53 penile cancer samples and found that 20 samples were positive for HPV-DNA. High-risk HPV-16 was the most common type of HPV (15/20). At the same time, the expression of p16^Ink4a^ was significantly increased in 13 of 15 HPV-16 samples (*P* < 0.01) (29). There was a close relationship between HPV infection and abnormal expression of p16^Ink4a^, which promotes the use of p16^Ink4a^ immunostaining as HPV detection. Additionally, Barzon et al. have analyzed microRNA (miRNA) of 59 patients with penile squamous cell carcinoma and found that the expression of miR-218 in high-risk HPV infection-positive samples was reduced (30). The decrease in miR-218 expression might be an important factor in HPV-induced carcinogenesis. Therefore, the use of condoms and HPV vaccination are essential. However, recommendations for HPV vaccination may vary from country to country.

Chronic inflammation is considered to be the carcinogenic mechanism of many malignant tumors. A total of 45% of penile cancer patients have a history of balanitis foreskin compared to only 8% of patients in the control group (31). A meta-analysis of 443 patients has found that the odds ratio (OR) of penile cancer with balanitis was 3.82. Similarly, lichen sclerosis (a type of chronic inflammation) and balanitis xerotic organisms (BXO) are also associated with the progression of penile cancer (32). Recent literature has shown that 28%–50% of patients with penile cancer have a history of BXO, and the estimated risk of developing penile squamous cell carcinoma is 2%–15% (33). This risk is mediated by the formation of phimosis, a known risk factor for penile cancer. Therefore, circumcision is necessary to prevent penile cancer, especially for patients with phimosis. The protective mechanism of circumcision is believed to improve hygiene, reduce the risk of transmission of HPV and other viruses, and reduce the occurrence of chronic inflammation. A recent systematic review by Larke et al. has found that early circumcision has a strong protective effect on invasive penile cancer (OR = 0.33) (34). Additionally, smoking, genital warts, age, obesity, metabolism, and social factors might be one of the risk factors for penile cancer.

#### 4.2.2 Surgical treatment for penile cancer

Surgery is the gold standard for the treatment of penile cancer (35). However, the penis is a male sexual organ. Due to social, psychological, physiological, and other factors, the surgical treatment of penile cancer should follow the principle of removing the focus while minimizing the damage to the penis while maintaining the original shape and function of the penis as much as possible (9). The lesions only limited to the foreskin or penis head or tumors before the T1 stage can be circumcised or locally removed, and close observation and follow-up are required after the operation. Phase T1 tumors limited to the penis and without lymph node metastasis can be partially resected. If the invasive penile cancer or tumor invades more than 1/2 of the length of the whole penis, total penis resection should be performed (36). Compared with the operation of penis preservation, total penis resection can better clear the lesions, but it often leads to the loss of the patient’s sexual function, accompanied by various psychological diseases that affect the patient’s social life, and seriously reduces the quality of life. The main route of metastasis of penile cancer is lymph node metastasis. In 1977, Cabanas first proposed the concept of penile sentinel limp nodes (SLN) (37). SLN is located in the groin region, which is the earliest lymph node of penile cancer metastasis. Therefore, it is very important to determine whether there is tumor cell metastasis in SLN of penis. At present, the evaluation of lymph nodes requires careful physical examination first (38). If the swollen lymph node is not touched, fine needle aspiration cytology (FNAC) is a less invasive and more accurate method to determine suspicious lymph node metastasis, or with CT/MRI and other imaging techniques (39). If it is positive, lymph node dissection should be performed, including open inguinal lymphadenectomy (OIL) and video endoscopic inguinal lymphadenectomy (VEIL). OIL has many complications, mainly including wound infection, skin necrosis, wound dehiscence, lymphedema, etc (40). Compared with OIL, VEIL has fewer postoperative complications and similar efficacy. It may be used as a first-line treatment for inguinal lymph node dissection in the future, but it still needs to be further confirmed by multi-center randomized controlled trials with higher quality and larger samples (41).

At present, in addition to surgical treatment, chemotherapy, radiotherapy, immunotherapy and other systematic therapies for penile cancer are also the focus of research in this field (42). National Comprehensive Cancer Network (NCCN) and European Association of Urology (EAU) guidelines recommend a multimodal first-line approach involving neoadjuvant chemotherapy (NAC) followed by lymph node dissection for the treatment of bulky nodal disease (43). In a prospective study (44), NAC was significantly associated with an improvement in overall survival and time to progression among responders versus non-responders (*P* < 0.01), and 30% of the cohort remained free of disease recurrence following a median follow-up period of 34 months. Pathologically, 50% of patients responded to NAC and 10% of patients showed a pathological complete response. In the setting of locally advanced penile cancer, current NCCN guidelines recommend adjuvant chemotherapy in the form of either TIP or 5-fluorouracil (5-FU) in patients who did not receive first-line NAC and exhibited ≥2 positive nodes or extranodal extension at the time of inguinal lymph node dissection (45). The debate on whether to use radiotherapy, surgery, or both in penile cancer management has been ongoing for more than 50 years (46). At present, data from randomized controlled trials comparing radiotherapy and surgery are lacking, and thus management is frequently determined by institutional practice patterns and available expertise. In recent years, more new systemic treatment options for penis cancer have been reported, including immune checkpoint inhibitors (ICIs) (47), adaptive and engineered T-cell therapies (48), tyrosine kinase inhibitors (TKIs) and other targeted therapies (49), and HPV targeting vaccines (50). However, most of the relevant types of literature are relatively small, and we will update this study accordingly after the data is enriched in the future.

The significance of our research is as follows. (1) The countries/regions, institutions, authors, journals, references, keywords, and other elements of penile cancer-related literature in the past 20 years were clearly displayed. (2) Through the collation of data sets, readers and experts in relevant fields were helped to understand the development process, research status, and knowledge hotspots of penile cancer. (3) Future research directions for penile cancer were further provided for reference. However, there were still some limitations to our study. 1. Recently published articles might have low citations due to the limited time available for citations; thus, the study might be prone to research bias. 2. This study only included articles and reviews published in English, which might have overlooked some of the literature. 3. With the rapid development of big data, this study might have a short timeframe and needs to be updated regularly.

## 5 Conclusion

In the past 20 years, the number of papers published in research related to penile cancer has shown a linear upward trend. Especially in the past five years, the number of papers published each year exceeded 110. The USA and Europe were leading in research on penile cancer. However, there is a need to strengthen collaboration relationships, especially in developing countries. The top 10 cited and co-cited journals were mostly in Q1 and Q2, reflecting that research on penile cancer was of high quality. Furthermore, this bibliometric analysis revealed that the main research topics and hotspots in penile cancer included risk factors and surgical treatment.

## Data availability statement

The raw data supporting the conclusions of this article will be made available by the authors, without undue reservation.

## Author contributions

SD and JW designed the study. SD, ZX and JF conducted the literature search. HL, BW, ZY, LX, FM and LW analyzed the data and wrote the paper. YX and JW approved the final manuscript. All authors contributed to the article and approved the submitted version.
